# Annual acknowledgement of manuscript reviewers

**DOI:** 10.1186/1550-2783-11-1

**Published:** 2014-01-17

**Authors:** Jose Antonio, Douglas Kalman, Richard Kreider

**Affiliations:** 1 Exercise and Sports Science, Nova Southeastern University, Davie, FL, 33314, USA; 2 Miami Research Associates, Endocrinology & Nutrition Department, 6141 Sunset Drive - Suite 301, Miami, FL 33143, USA; 3 Exercise & Sport Nutrition Lab, Department of Health & Kinesiology, Texas A&M University, College Station, TX, USA

## Abstract

**Contributing reviewers:**

The editors of Journal of the International Society of Sports Nutrition would like to thank all our reviewers who have contributed to the journal in Volume 10 (2013). The tireless work that all of you have given to us to enhance our journal is highly appreciated. We can’t thank you enough.

## 

Michelle Adams

United States of America

Jose Antonio

United States of America

George Aphamis

Cyprus

Alan Aragon

United States of America

Todd Astorino

United States of America

Michelle Barrack

United States of America

Pedro Bastos

Portugal

Jenna Becker

United States of America

Dan Benardot

United States of America

Sandeep Bhale

United States of America

Wilhelm Bloch

Germany

Leigh Breen

United Kingdom

Aurelien Bringard

Switzerland

Grant David Brinkworth

Australia

Luke Bucci

United States of America

Bill Campbell

United States of America

Erico Caperuto

Brazil

Amanda Carlson-Phillips

United States of America

Michael Carlston

United States of America

Amelia Carr

Australia

Chantal Charo

United States of America

Hamdi Chtourou

Tunisia

Amanda Claassen-Smithers

South Africa

Pablo Costa

United States Minor Outlying Islands

Paul Cribb

Australia

Maria Daglia

Italy

Vincent Dalbo

Australia

Lance Dalleck

New Zealand

Barbara Dao

Canada

Ben Dascombe

Australia

Patrick Davitt

United States of America

Jay Dawes

United States of America

Felipe Donatto

Brazil

Inna Dumova

United States of America

Christopher Dunbar

United States of America

Travis Dutka

Australia

Joan Eckerson

United States of America

Chris Fahs

United States of America

Andrew Foskett

New Zealand

David Fukuda

United States of America

Jeffrey Godin

United States of America

Joanna Gromadzka-Ostrowska

Poland

G. Gregory Haff

Australia

Travis Harvey

United States of America

Marvin Heuer

United States of America

Matthew Higgins

United Kingdom

Ralf Jäger

United States of America

Jørgen Jensen

Norway

Andrew Jones

United Kingdom

Elizabeth Joseph

United States of America

Douglas Kalman

United States of America

Lynne Kammer

United States of America

Chad Kerksick

United States of America

Liam Kilduff

United Kingdom

Anil Kulkarni

United States of America

Paul La Bounty

United States of America

Warren Ladiges

United States of America

Antonio Lancha

Brazil

Hubert Lee

United States of America

Sukho Lee

United States of America

Anna Lepeley

United States of America

Michael Lever

New Zealand

Hector Lopez

United States of America

Lonnie Lowery

United States of America

Ryan Lowery

United States of America

Shannan Lynch

United States of America

Ian Macdonald

United Kingdom

Maria Rita Marques de Oliveira

Brazil

Linda McCargar

Canada

Andrew McKune

South Africa

Goncalo Mendonca

Portugal

Ricardo Mora-Rodriguez

Spain

Carolina Moura

Brazil

Michael Nelson

United States of America

Georg Neumann

Germany

Layne Norton

United States of America

Michael Ormsbee

United States of America

Corey Peacock

United States of America

Peter Peeling

Australia

Dungeng Peng

United States of America

Mike Price

United Kingdom

Peter Res

Netherlands

Todd Rideout

United States of America

Robert Robergs

Australia

Michael Roberts

United States of America

Judith Rodriguez

United States of America

Brian Roy

Canada

Eric Ryan

United States of America

Craig Sale

United Kingdom

Sylvia Santosa

Canada

Timothy Scheett

United States of America

Steve Schmitz

United States of America

Brad Schoenfeld

United States of America

Christopher Scott

United States of America

Richard Seip

United States of America

Piero Sestili

Italy

Andrew Shao

United States of America

Jason Siegler

Australia

Tobin Silver

United States of America

Carsten Smidt

United States of America

Laura Stewart

United States of America

Robert Takla

United States of America

Minghua Tang

United States of America

Yuko Tanimura

Japan

Lemuel W Taylor

United States of America

Jay Udani

United States of America

Jeff Volek

United States of America

Xiao Wan

China

Benjamin Wax

United States of America

Joseph Weir

United States of America

Shawn Wells

United States of America

Louise Weschler

United States of America

Rob Wildman

United States of America

Boguslaw Wilk

Canada

Mel Williams

United States of America

Gabriel Wilson

United States of America

Gordon Zello

Canada

Yang Zhang

China

Tim Ziegenfuss

United States of America

